# Modulating microRNAs in cancer: next-generation therapies

**DOI:** 10.20892/j.issn.2095-3941.2021.0294

**Published:** 2021-12-01

**Authors:** Nahid Arghiani, Khalid Shah

**Affiliations:** 1 Center for Stem Cell and Translational Immunotherapy (CSTI), Harvard Medical School, Boston, MA 02115, USA; 2 Department of Neurosurgery, Brigham and Women’s Hospital, Harvard Medical School, Boston, MA 02115, USA; 3 Harvard Stem Cell Institute, Harvard University, Cambridge, MA 02138, USA

**Keywords:** MiRNAs, dysregulation, delivery systems, cancer therapy, clinical translation

## Abstract

MicroRNAs (miRNAs) are a class of endogenously expressed non-coding regulators of the genome with an ability to mediate a variety of biological and pathological processes. There is growing evidence demonstrating frequent dysregulation of microRNAs in cancer cells, which is associated with tumor initiation, development, migration, invasion, resisting cell death, and drug resistance. Studies have shown that modulation of these small RNAs is a novel and promising therapeutic tool in the treatment of a variety of diseases, especially cancer, due to their broad influence on multiple cellular processes. However, suboptimal delivery of the appropriate miRNA to the cancer sites, quick degradation by nucleases in the blood circulation, and off target effects have limited their research and clinical applications. Therefore, there is a pressing need to improve the therapeutic efficacy of miRNA modulators, while at the same time reducing their toxicities. Several delivery vehicles for miRNA modulators have been shown to be effective *in vitro* and *in vivo*. In this review, we will discuss the role and importance of miRNAs in cancer and provide perspectives on currently available carriers for miRNA modulation. We will also summarize the challenges and prospects for the clinical translation of miRNA-based therapeutic strategies.

## Introduction

Cancer is a major global health concern that imposes a significant financial burden. Despite advances in cancer treatment, new therapeutic approaches are needed. Targeting molecules that have key regulatory functions on up to 200 mRNAs and multiple pathways may eventually provide an efficient cancer cure. Increasing evidence shows that miRNAs, which are small untranslated molecules, can regulate multiple genes important in cancer formation and development. These small RNAs have emerged as highly conserved non-coding endogenous RNAs in 142 species, and have been implicated as key regulators of different biological processes, mostly through gene-silencing pathways^1,2^. MiRNAs can target gene expression *via* direct interaction with DNA, translational repression, or mRNA degradation. Thus, the biogenesis and expression of miRNAs are carefully controlled by multiple molecular mechanisms; a certain set of miRNAs are expressed in specific cell types during particular stages in development^3^. Furthermore, aborted expression of miRNAs is involved in initiation, development, and progression of different diseases including cancer^4^. The therapeutic potential of miRNA modulation is supported by its use in treating different cancer types in preclinical models. Recently, research groups have focused on optimizing strategies for miRNA delivery to tumor sites, as well as reducing their toxicities to normal cells in order to accelerate clinical translation. Here, we first review the role of miRNAs in cancer and shed a light on the current status and recent progress of therapeutic miRNA delivery systems. We also discuss the challenges and advances in miRNA-based therapeutics, leading to a number of clinical trials.

## Dysregulation of microRNAs in cancer

Oncogenic miRNAs can be classified into 2 groups based on expression levels. Generally, miRNAs that are overexpressed in cancer cells that promote tumorigenesis by inhibiting tumor suppressors are known as “oncomiRs,” whereas down-regulated miRNAs that prevent tumor development by inhibiting the expressions of oncogenes are known as “tumor suppressor miRs.” Dysregulation can occur in a single oncogenic microRNA or global miRNA expression through various mechanisms. Upregulation of oncomiRs has been shown to result in a gain-of-function mutation, hypomethylation, gene amplification, or aberrant expression of oncomiRs^5^.

Furthermore, evidence from multiple sources has shown that downregulation of tumor suppressor miRs is the consequence of their deletion or inactivation through mutation or heterochromatin formation. In such cases, epigenetic alterations such as aberrant DNA methylation and histone modifications play important roles^5^.

Additionally, aborted expression of miRNAs can be associated with aberrant activity of transcription factors such as MYC, TP53, STAT3, SP1, and RREB1^6,7^. For example, elevated expression of c-MYC induces increased levels of oncogenic miR-155 and miR-17–92 clusters *via* interaction with the E-box in the promoter region of these miRNAs. In addition, c-MYC induces cancer initiation through repression of tumor-suppressive microRNAs such as let-7, miR-23b and miR-29a^8,9^. Other studies have demonstrated that miRNAs such as miR-34 and miR-605 are regulated by P53, leading to cell cycle arrest and induction of apoptosis^10,11^. Disturbing the interactions of STAT3 and NF-kB with binding sites at the hsa-miR-21 promoter results in downregulation of this small RNA^12^. This study indicated that upregulation of miR-21 was regulated by STAT3 and NF-kB. Transcription factors like the zinc finger protein, RREB1, are activated by the RAS oncogene and bind to RAS-responsive element (RRE), which is located in the miR-143/145 cluster promoter. RREB1 activation leads to repression of miR-143/145 cluster expression in pancreatic cancers. In addition, expressions of KRAS and RREB1 are adversely controlled by miR-143/145^13^. The transcriptional repressor ZEB1 also inhibits the expression of miR-141 and miR-200c *via* binding to the ZEB-type E-box element located within target gene promoters. ZEB1 causes the epithelial-to-mesenchymal transition (EMT) in cancer cells^14^. Increasing evidence also suggests that global changes in miRNA expression reflect defective biogenesis systems. Copy number abnormalities or deregulated expression of components involved in the miRNA pathway such as Drosha, DGCR8, Dicer, and TRBP have been related to promotion of cancers^15^.

Utilizing matched primary and metastatic tumor pairs and robust metastasis models, miRNAs have been shown to be the main regulatory components of molecular networks during metastasis. MetastamiRs refer to those regulatory miRNAs, which promote or inhibit invasion and metastasis of cancer cells through regulation of key steps in the metastatic program such as cancer stem cell properties, EMT, adhesion and proteolysis, and migration^16^. Stemness is responsible for initiating metastasis and cancer recurrence, and miRNAs are involved in the maintenance of the cancer stem cell *via* targeting main signaling pathways such as WNT/β-catenin, NOTCH, NF-kB, PI3K/AKT/mTOR, BMI-1, and STAT3. These pathways are targeted by many families such as miR-7, miR-10b, miR-21, miR-31, miR-140-5p, and miR-34a^17^. Additionally, studies have shown miR-200 and miR-205 increased the EMT by increasing the expression of ZEB1. In contrast, the forced expression of miR-1199-5p significantly decreases ZEB1 and hinders EMT^18^. Dysregulation of miR-101 and miR-9 has been shown to induce metastasis by activating Zeste Homolog 2 (EZH2) and the β-catenin pathway, as well as by targeting the EMT *via* targeting E-cadherin^19^.

A growing body of evidence indicates that several miRNAs participate in cancer cell resistance by directly or indirectly regulating different signaling pathways^20^. MicroRNAs seem to reduce the effectiveness of treatments by regulating the cell cycle, DNA damage repair systems, intake of anticancer drugs and drug efflux, apoptosis resistance, drug inactivation, and suppression of intracellular prodrug activation. For example, miR-34a, miR-124, miR-106a, miR-188, miR-195 miR-449a, and miR-320c can affect cell cycle progression and the sensitivity of cancer cells by targeting CDK, while miR-192, miR-221, and miR-223 are involved in radiation and chemotherapy resistance *via* regulating the cell cycle by targeting p27^21^. Furthermore, the upregulation of miR-21, miR-27a, and miR-451 contributes to overexpression of P-glycoprotein, a major efflux pump in humans, whereas miR-138 and miR-298 increase the sensitivity to anticancer drug resistance of the MDR cell line in leukemia and breast cancer^22^. A large number of miRNAs, such as miR-9, miR-19a, miR-19b-3p, miR-21, miR-125b, miR-192, miR-221, and miR-222 regulate core apoptotic signaling pathways and play key roles in radiotherapy and drug resistance *via* targeting of PTEN, BCL-2, PARP, and PDCD4 in different cancer cells^23,24^. It is well-known that miR-21, miR-122–5p, miR-132–5p, miR-320, and miR-892a alter expressions of enzymes involved in intracellular activation of some chemotherapeutic agents and/or Cytochrome P450 (P450)-related enzymes, which are important for the clearance of toxic drugs^25^. Additionally, down-regulating miR-135a, miR-23a, and miR-203 can increase IL-17 and IL-8 and activate STAT3, which has a negative impact on the outcomes of radiotherapy^20,24^.

Overall, changes in miRNA levels have potential value in clinical applications as effective diagnostic and prognostic markers or predictive markers of responses to treatments. In different studies, profiles of miRNA expressions have been used to identify cancer types and stages. In addition, correlations between miRNA signatures and efficacies of responses to different therapeutic regimens have great clinical value^26^. Understanding the molecular mechanisms involved in miRNA dysregulation could therefore be useful in selecting appropriate target molecules or treatment approaches.

## The role of microRNAs in the tumor microenvironment

**Figure 1:**
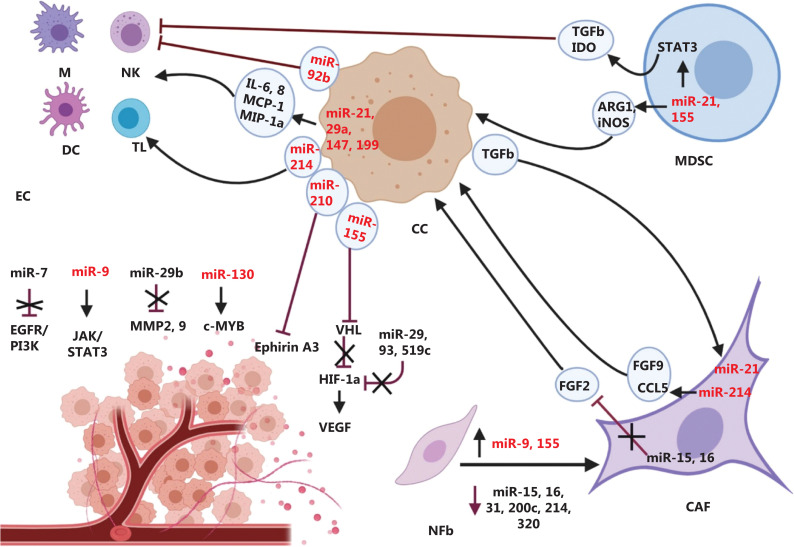
Dysregulation of miRNAs in tumor microenvironment. MiRNAs mediate crosstalk between tumor cells and their microenvironment. Red and black colors represent oncomiRs and tumor suppressor miRNAs, respectively. ARG1: Arginase 1; CAF: Cancer associated fibroblast; CC: Cancer cell; DC: Dendritic cells; EC: Endothelial cell; IDO: Indoleamine 2,3-dioxygenase; iNOS: inducible NO synthase; M: Macrophage; MDSC: Myeloid-derived suppressor cell; NFb: Normal fibroblast; NK: Natural killer cells; TL: T lymphocyte.

The environments surrounding tumors are recognized as key contributors to tumor growth, angiogenesis, metastasis, and therapeutic resistance^27,28^. Several studies have shown that molecular interactions among tumor cells and other cell types such as endothelial cells (ECs), fibroblasts, and innate and adaptive immune cells in the tumor microenvironments regulate tumor development^29^. MiRNAs can participate in intracellular communication as mediators of signaling by amplifying a signal or tuning its response (**Figure 1**). However, they are also released by cells and mediate crosstalk between tumor cells and their microenvironments^30^. Previous studies have demonstrated that miRNAs have important effects in transdifferentiation of normal fibroblasts to cancer-associated fibroblasts (CAFs). For example, dysregulation of miRNAs like miR-9, miR-21, miR-15, miR-16, miR-31, miR-155, miR-200s, miR-320, and miR-214 reprogram fibroblasts to CAFs^31^. In addition, tumor-secreted factors such as TGF-β are known to influence miRNA expression in CAFs. For example, miR-21 expression is upregulated in CAFs by cancer cells secreting TGF-β, which results in uncontrolled proliferation of cancer cells^32^. In addition, miRNAs can affect chemokines and growth factors derived from CAFs, to participate in cancer cell invasion and metastasis. In such cases, low expression of miR-214 in the tumor microenvironment increases chemokine (C-C motif) ligand 5 and fibroblast growth factor 9 (FGF9), which contributes to tumor growth and motility^33^. Furthermore, significant downregulations of miR-15 and miR-16 have been reported in CAFs, which promotes proliferation and migration in cancer cells through activation of the FGFR1 pathway^34^.

MiRNAs also play key regulatory roles in pathways related to angiogenesis within the tumor microenvironment. Some of the most commonly known miRNAs, such as miR-93, miR-29, and miR-519c negatively control angiogenesis while miR-155 and miR-210 can positively influence this complex process^35–37^. During hypoxia, the overexpression of miR-130a has been shown to promote endothelial cell migration *via* targeting c-MYB^38^. Other studies have also revealed that miR-21 can induce angiogenesis *via* activation of the AKT and ERK1/2 signaling pathways, which elevate levels of VEGF and HIF-1α^39^. MiR-9 can stimulate EC migration and angiogenesis *via* aberrantly activating the JAK-STAT pathway^40^. Furthermore, miR-7 has emerged as an inhibitor of angiogenesis by targeting the EGFR/PI3K/AKT signaling pathways^41^. Moreover, miRNAs like miR-29b reduce expression and activity of ECM signaling genes such as MMP2 and MMP9. Thus, downregulation of this miRNA attenuates angiogenesis through the MAPK/ERK and PI3K/AKT signaling pathways, directly affecting cancer metastasis^42^.

Diverse infiltrated immune cells are present in the tumor microenvironment. Previous studies have revealed that miRNAs also affected the immune cells recruited to cancer sites. For example, miR-155 and miR-21 are mostly upregulated in myeloid-derived suppressor cells (MDSCs), and promote immunosuppressive effects *via* activation of the STAT3 pathway in cancer cells^43^. These miRNAs can induce MDSC expansion, which contributes to tumor progression. Conversely, reduction of the anti-inflammatory miRNA, miR-199a, leads to activation of the NF-kB signaling pathway and tumor progression^44,45^. Previous studies have also shown that miRNAs such as miR-21, miR-29a, and miR-147 released from cancer cells can regulate the inflammatory response and result in cancer progression and invasion^46–48^. Nakano and co-workers reported that cancer cell-derived exosomal miR-92b inhibited natural killer (NK) cell-mediated cytotoxicity by deregulating CD69^49^. Furthermore, transfer of cancer cell-secreted miR-214 to regulatory T cells (Tregs) suppresses immune responses at the tumor site *via* promoting Treg expansion^50^. Taken together, given the pivotal roles of miRNAs in the tumor microenvironment, targeting these small molecules presents a very promising strategy for cancer treatment.

## Therapeutic strategies for microRNAs

Several methods have been characterized to find appropriate target miRNAs for the most effective cancer treatments based on the cancer type. Among them, a combination of miRNA sequencing (miRNA-seq) and bioinformatics analysis has been shown to facilitate the identification of miRNAs. MicroRNA sequencing has also been used to quantitatively profile small RNAs and discover new miRNAs, as well as investigate their functions^51–54^. Recently, Wang and co-workers have shown that co-sequencing microRNAs and mRNAs in the same cell revealed the non-genetic heterogeneity of small RNAs, while at the same time improving our understanding of the contribution of different pathways in controlling miRNA expression^55^.

Different strategies have been reported for regulating miRNA expression, 1 of which is restoration of suppressed miRNA levels using synthetic miRNA mimics or miR-expressing vectors. Other strategies developed to suppress oncomiRs include using anti-miR (AMOs), miRNA sponges, miRNA masking, and small molecule inhibitors (SMIRs) that can epigenetically influence miRNA expression^56,57^. AMOs are chemically modified synthetic antisense sequences, and despite their transient inhibition due to dilution during cell replication, they are still the most popular and are frequently used for their simple structures. The miRsponge consists of multiple tandem complementary binding sites designed to trap a specific miRNA, whereas miRNA masks are single-stranded antisense oligonucleotides designed to block the desired miRNA target site in specific mRNAs^58,59^. In contrast, SMIRs can block miRNA biogenesis at the pre-transcription, transcription, and post-transcription levels. They offer some advantages such as short production time, low cost, and long circulation times in the blood^60^. However, SMIRs may have off-target effects in healthy tissues^58,59^. Recent studies have also shown that CRISPR/Cas9, a feasible and simple strategy for creating gene knockout or knock-in human cells, could also efficiently generate miRNA knockout mutants^61^. All of these strategies are more efficient *in vitro* than *in vivo* due to insufficient *in vivo* delivery of these molecules. Therefore, it is necessary to identify better delivery systems and proper administration routes to overcome this limitation.

**Figure 2:**
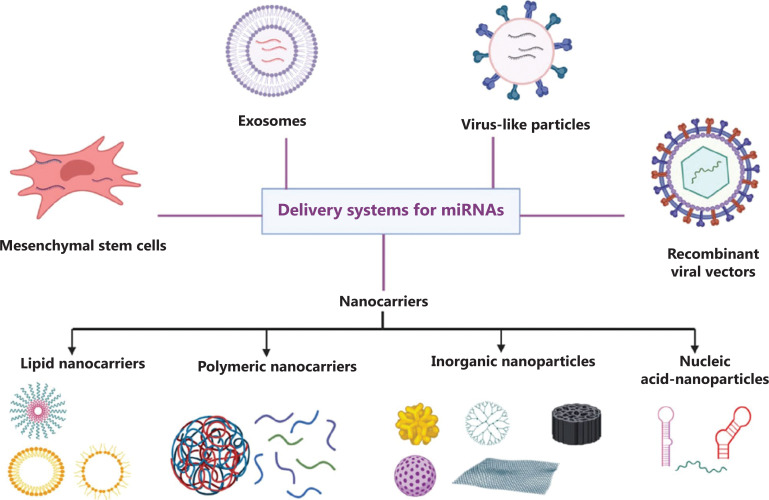
Schematic illustration of different vehicles applied for miRNAs delivery to cancer cells. The 5 major groups of microRNA delivery systems are nano delivery systems, viral vectors, virus-like particles, exosomes and mesenchymal stem cells that have their own advantages and disadvantages. Choosing delivery systems based on the type and stage of cancer might accelerate translating miRNA-based therapeutics into the clinic.

The major miRNA administration routes are local and systemic administration. Compared with systemic administration, local administration has shown fewer side effects and toxicity. In addition, local delivery seems to be a promising approach to robustly protect small RNAs from endogenous RNase, and requires only a minimal dose of these molecules to obtain maximum therapeutic effects^62–64^. Although systemic administration is more effective in metastatic cancers, rapid clearance from the body is still an issue^63^. Additionally, negatively charged hydrophilic miRNAs have difficultly passing through the cell membrane and penetrating cells. Thus, for maximizing the killing potential of miRNA, carrier systems must protect small RNAs from degradation, avoid rapid clearance, and increase their circulation time in the blood stream. This would facilitate their specific uptake by cancer cells with less toxicity to normal cells. Several microRNA carrier systems that have recently been developed (**Figure 2**) are briefly discussed below.

### Nanoparticle-based microRNA delivery systems

Although nanocarrier delivery systems are a relatively new area of research, they are rapidly establishing their own niche in the pharmaceutical industry due to their small sizes and low molecular weights. Nanoparticle-based microRNA delivery systems are divided into 4 major groups, each of which has its advantages and disadvantages, involving lipid-based carriers, polymer-based delivery systems, inorganic nanoparticles, and nucleic acid-based delivery systems (**Table 1**). Most of these nanoparticles can be functionalized by conjugating with antibodies.

**Table 1:** Comparison of various nanocarriers for miRNA-based therapy

Delivery system	Advantage	Disadvantage	Ref.
Lipid-based systems			
Cationic lipids	High transfection efficacy, reduced rate of phagocytic clearance, easy large-scale production	Accumulation of particles in liver, spleen and lung, interferon response induction, possible elimination by mononuclear phagocyte system	^65–67^
Neutral lipids	Less cytotoxic effects, non-immunogenic, non-phagocytic elimination	Low loading capacity and transfection efficacy for miRNAs, hardly endocytosed by cells	^68–70^
Ionizable lipids	Limited side effects, non-immunogenic, longer circulating time	Low loading capacity	^65,71^
Polymer-based systems			
Chitosans	pH tunable drug releasing system, low immunogenicity, mucoadhesive and antibacterial potential	Poor stability, less solubility, low transfection efficacy and lack of control over pore-size property	^72,73^
Dendrimers	Good stability, easily modified at the surface	Hemolytic activity, uncontrolled release of drug	^74,75^
PLGAs^a^	Biocompatibility, controllable release of drug, prolonged residence time in vital organs	Poor drug loading, high production costs, difficult to scale-up	^76–78^
PEIs^b^	High buffering and loading capacities	Toxicity, poor biodegradable polymer	^79,80^
Poly lysines	Slow degradation and gradually release of drugs	High charge density, toxicity	^81–83^
Protamines	Improves delivery of siRNAs	Associated with some side effects such as pulmonary hypertension and anaphylactic	^84,85^
CPPs^c^	Easy preparation, reserving biological activity of cargo, low cytotoxicity	Heterogeneity of the nanoparticle, interaction with plasma protein, low *in vivo* efficacy	^82,86,87^
R3V6	Transportation of small RNAs more effective than PEI and lipofectamine	-	^88,89^
Atelocollage	Reduced cargo immunogenicity, high transfection efficiency	Possible immunogenicity	^90–92^
Inorganic nanoparticles			
Golden nanoparticles	Easily modified at the surface, high stability, non-immunogenic, controllable drug loading and release deep inside tissues	Less drug loading capacity	^93,94^
MSNs^d^	Non-toxicity, high drug loading capability, easily modified at the surface, tunable pore structures, releasing agents in response to specific signals	Production and reproducibility problems in large scales	^95^
IONPs^e^	Easy preparation, biocompatible, low toxicity, high stability	Very long circulation time	^96^
QDs^f^	Strong adsorption capacity, more reactivity activity, smaller size	Immune response induction when using heavy metals for preparation of QDs	^97–100^
GOs^g^	Antibacterial properties, low toxicity, easier translocation across the membrane	Require more studies to prove the biocompatibility of GO *in vivo*	^101,102^
NFs^h^	Low cost, controlled releasing of drug over a definite period, more feasibility to load miRNAs for long-term delivery application	Limited control on pore size of particles, become brittleness after calcination	^103–107^
Folate	Quickly taken up by cancer cells, easier penetration of miRNA to dense extracellular matrix in solid tumors		^108^
Nucleic acid-based delivery systems			
Aptamers	High safety, high binding affinity to target cells	Easy degradation by blood nuclease, difficulties in conjugating with some therapeutic agent	^109,110^
pRNA^i^	High solubility and stability, long half-life		^111–113^

^a^Poly(lactic-co-glycolic acid); ^b^polyethyleneimines; ^c^cell-penetrating peptides; ^d^mesoporous silica nanoparticles; ^e^iron oxide nanoparticles; ^f^quantum dots; ^g^graphene oxide; ^h^nanofibers; ^i^packaging RNA.

#### Lipid-based carriers

Lipid nanocarriers are made of both solid and liquid lipids by homogenization-emulsification methods to encapsulate different drugs^114^. Three groups of lipids (cationic, neutral, and ionizable) are commonly used for delivery of miRNAs to cancer cells^65^. Recently, ionizable lipids have been used more often than cationic and neutral lipids for safe and efficient *in vivo* delivery. At acidic pH, negatively charged miRNAs are encapsulated in pH-sensitive ionizable lipids *via* electrostatic interactions. Because these particles have essentially no surface charge at near-physiological pH, they are less likely to form aggregates with plasma proteins and do not induce a robust immune response^65^. In the acidic tumor microenvironment, positively charged ionizable lipid particles can easily bind to negatively charged cell membranes, increasing cellular uptake. Thus, these smart lipid particles are suitable for systemic and local administration of drugs and can reduce undesirable side effects^115–117^. However, a higher loading capacity and modifications of the lipids are needed to translate these lipid carriers into the clinical setting.

#### Polymer-based systems

Polymer-based delivery systems are divided into natural and synthetic groups. Abundant natural polymers like chitosan possess mucoadhesive properties, have low toxicity, and have pH-tunable drug release characteristics, making them ideal candidates as drug carriers^73,118^. Other natural polymers, such as cell-penetrating peptides (CPP) can pass through cellular membranes to deliver a variety of biologically active conjugates^87^. However, despite the higher stability, good cellular uptake and lower toxicity of CPP-conjugated nanoparticles, they may increase particle aggregation and endosomal entrapment^86^. Synthetic polymers include dendrimers, poly(lactic-co-glycolic acid) (PLGA), and polyethyleneimines (PEIs). Although synthetic polymers are easily modifiable and have high buffering capacities, their toxicity and uncontrolled release of drug still need to be addressed^44,76,77,79,119^.

#### Nucleic acid-based delivery systems

DNA nanotechnology has shown great potential for preparing structures that serve as delivery systems. The advantages of using nucleic acids as delivery vehicles as compared to peptide-based delivery systems include lower induced immunity, higher solubility, greater thermodynamic stability, and lower toxicity. Aptamers and pRNAs are 2 classes of these carriers. Aptamers are a group of nucleic acid delivery vehicles often called chemical antibodies, which are capable of binding various molecules with high affinity and specificity. These molecules can serve as cancer therapeutics as well as diagnostic and delivery tools^109^. To increase drug-loading capacity, a polyvalent aptamer system composed of Mucin1 and AS1411 with pH-sensitive drug-release properties was developed^120^. Although aptamers have rapidly advanced the drug delivery field, some limitations that reduce their efficacy still need to be addressed, such as degradation in the presence of blood nucleases and nonspecific interactions with bloodstream proteins^109^. Previous studies have shown that packaging RNA (pRNA), a conserved noncoding RNA in the phi29 DNA packaging motor, has a strong tendency to form multimeric structures and therefore represents an ideal drug delivery system for several therapeutic agents^121,122^. Additional nuclease stability is conferred to this RNA by chemical modifications using 2′-fluoro on the ribose sugar of nucleotides^111^. Furthermore, to enhance the mechanical and thermal stability of these carriers, the pRNA contains an ultra-stable three-way junction (3WJ), which was developed for successful translation of small RNAs to cancer cells^111,123^.

### Viral-based microRNA delivery methods

Synthetic viral vectors are emerging as promising tools for gene therapy due to their high transduction efficacy and long-lasting gene expression in different types of cells. The viruses most commonly utilized for delivery of therapeutic genes are lentiviruses (LVs), adenoviruses (Ads), and adeno-associated viruses (AAVs)^63,124,125^. HIV-based vectors have been developed to deliver therapeutic genes, although other LVs including SIV and FIV are also being explored^87,126^. LVs have also been used for microRNA replacement therapy^63^. However, insertional mutagenesis, genetic instability, activation of oncogenes, and histone modifications may occur after integration of the retroviral genome into the host cell genome, limiting their clinical use^78,127–129^. To overcome the risk of insertional mutagenesis in cytotoxic cancer therapies, integration-defective lentiviral vectors have been developed using mutations in the viral integrase or attachment sites. For example, overexpression of miR-145 using episomal lentiviral vector can stop proliferation and induce apoptosis in esophageal cancer cells^130^.

Third-generation adenoviruses can express miRNAs in a stable manner without integrating into the host genome^131^. In 1 study, engineered oncolytic adenoviruses were used to deliver miRNA-148a to pancreatic cancer cells without risking viral-associated toxicity in normal cells^132^. However, a major drawback of these vectors is that they strongly induce immune responses. To overcome this problem, adenoviruses have been chemically or physically modified using polymers. In addition, to enhance their tumor tropism, a variety of ligands have been introduced into coated-Ads^133,134^. AAVs also have good safety and display low immunogenicity, and their wide tissue tropism makes them promising tools for gene delivery *in vivo*^135,136^. So far, there are more than 13 AAV serotypes identified, most of which have been tested in more than 200 gene delivery trial studies^135,137^. Though limited insert capacity is the most familiar disadvantage of AAVs^138^; they remain good candidates for delivery of small DNA such as miRNAs and siRNAs. Bhere et al. showed that co-delivery of miR-21 and miR-7 mediated by AVVs effectively induced apoptosis in mice bearing brain tumors^56^. Additionally, AAV-miRNAs can also be used in combination with other therapeutic methods. In a study characterizing the therapeutic potential of miR-7-AAVs and S-TRAIL-MSCs for glioblastomas, the delivery of miR-7 *via* AAV led to upregulation of death receptor5 (DR 5), also known as TRAIL. Co-delivery of miR-7-AAVs and S-TRIAL-MSCs was shown to result in strong tumor growth inhibition and prolonged mouse survival^139^. Overall, delivery of AAV-mediated miRNAs holds great promise in cancer therapy. In summary, viral vectors provide long-term stable expression of the desired gene. They could become an efficient means of clinical application of miRNAs if their stimulation of immune responses can be overcome.

### Virus-like particle (VLP) delivery systems

VLPs, or noninfectious viral protein structures, were successfully developed for drug delivery in the early 1990s. VLPs can reassemble *in vitro*, after which they can be loaded with drugs or small molecules^140–142^. MS2 is a member of the Leviviridae family, with single strand-RNA that infects *Enterobacteriaceae*^143,144^. MS2 can be produced as pseudo-viral particles and possesses multiple unique features that makes it an interesting candidate for targeted gene delivery, such as quick production, self-assembly in the presence of nucleic acids, and easy capsid modification^145^. Several studies suggested that Tat modification of VLPs improved the cellular uptake of these particles. Delivery of miR-146a was increased 1−14-fold *in vitro* and 2-fold *in vivo* after Tat peptide grafting^146^. Likewise, VLPs derived from PP7 are nontoxic and protect RNAs against nucleolytic attack. Interestingly, they are more resistant to high temperatures than MS2. Sun et al. demonstrated that TAT peptide and pre-microRNA-23b co-loaded with PP7 VLP could be effectively delivered to hepatoma cells and significantly inhibited their migration^147^. Furthermore, it is well documented that the capsid protein (VP1) of the John Cunningham virus (JC), which belongs to the Polyomavirus family and causes central nervous disease, has self-assembling properties both *in vitro* and *in vivo*, and is useful in creating VLPs. The superiority of these VLPs over PP7 and MS2 VLPs for treating brain diseases is that VP1 mediates cellular entry through interaction with cell receptors in the central nervous system^148,149^. In brief, VLPs are thermoresistant and more stable than lipid-based particles, which protect miRNAs from rapid degradation by nucleases. The 2 most distinctive characteristics of these particles are their lower toxicities and infectivities due to their lack of a viral genome. The pH-dependent assembly-disassembly behavior of VLPs also makes them attractive candidates for drug delivery to target cells *via* exogenously produced virus structures. Although VLPs can be easily modified to specifically target cancer cells, overall use and successful clinical translation of these particles will depend on solving their immunogenicity problems^150–152^.

### Delivery of exosome-mediated microRNAs

Exosomes are nanosized cell-derived vesicles with a diameter range of 40−100 nm. These small particles originate from the endocytic pathway and are present in most body fluids. Exosomes can be taken-up by recipient cells, whose biological functions are altered in a paracrine manner. Because exosomes originate from the plasma membrane, they are safe and biocompatible, and induce immune tolerance *in vivo*^153–155^. An important feature of exosomes is their ability to overcome otherwise impermeable biological barriers such as the blood-brain barrier (BBB). Most previous studies used exosomes derived from mesenchymal stem cells (MSCs) and HEK cells as vehicles for loading miRNAs. Shimbo et al. demonstrated that MSC-derived exosomes containing synthetic miR-143 reduced the migration of osteosarcoma cells. However, the efficacy of exosome-mediated delivery of miR-143 was lower than that of lipofection^156^. One strategy to improve the pharmacokinetic properties of these small vesicles is to introduce appropriate ligands to specifically target cancer cells. In 1 study, Ohno et al. engineered donor cells to express the transmembrane domain of the platelet-derived growth factor receptor fused to the GE11 peptide, which specifically bound EGFR-expressing breast cancer cells. Their results showed that let-7a-containing GE11-exosomes suppressed the growth of xenograft breast tumor in mice^157^. In another study, exosomes derived from dendritic cells were modified with aptamer AS1411 to facilitate targeting of breast tumor cells^158^. Another strategy for increasing extracellular vesicle (EV) therapeutic efficacy is to enhance drug loading into these nanocarriers without affecting their membrane integrity. However, the inability to produce large quantities of highly purified EVs, as well as their rapid clearance from the bloodstream and accumulation in lung, liver, and spleen are major obstacles associated with this method^159–161^. Solutions to the drawbacks associated with the application of EVs as miRNA delivery systems are therefore needed before clinical studies can be initiated.

### MSC-based microRNA delivery

MSCs are 1 of the most popular types of stem cells used in clinical trials. Their immunomodulatory properties and suppression of inflammatory responses *in vivo* make them good candidates for stem cell-based therapy^162–164^. Furthermore, MSCs are easily engineered to improve their therapeutic potential or modify their adverse effects^165,166^. As small molecules can easily translocate between MSCs and various other cell types through gap junctions and exosomes, MSCs have been examined as carriers for therapeutic RNAs. Munoz et al. reported that delivery of anti-miR-9 to chemoresistant glioblastoma multiforme (GBM) cells sensitized them to temozolomide^167^. MiR-124 and miR-145 mimics were also delivered using MSCs to glioma cells and glioma stem cells *in vitro* and *in vivo*. The results of that study showed that delivery of miR-124 or miR-145 decreased both migration and self-renewal in GBMs^168^. Contradicting their results, our study indicated that co-culture of LV-anti-miR-21-Ad-MSCs with prostate, colon, and GBM cancer cells did not significantly inhibit tumor cell growth^56^. However, these contradictions may arise from the source from which the MSCs are derived or the enrichment of the desired miRNA in exosomes. Despite the many advantages of MSCs, the major drawback with using stem cells as biological carriers for cancer treatment is that tumor cells can produce factors that enhance proliferation and invasion of stem cells^169^. To overcome this shortcoming, it would be better to use a combination of stem cell-based delivery systems and other therapeutic approaches^170^.

## Clinical translation

As miRNAs are easily detected in body fluids, they could be used as noninvasive markers for early diagnosis of cancers. The first commercial miR-based diagnostic kit, miRNA-7™, was designed for liver cancer detection, and well-defined panels of miRNAs improved the sensitivity of cancer detection^170^. For example, ThyraMIR offers miR-based thyroid cancer screening and stratification based on the expression profiles of miRNAs such as miR-29b-1-5p, miR-31-5p, miR-138-1-3p, miR-139-5p, miR-146b-5p, miR-155, miR-204-5p, miR-222-3p, miR-375, and miR-551b-3p. ThyraMIR has been used in combination with ThyGeNEXT^®^ to distinguish between benign and malignant lesions^171^.

Clinical trials utilizing microRNA for cancer therapy have shown promising results. In 2013, the first report of miR mimics entering clinical trials involved MRX34^172^. MiR-34 is lost or down-regulated in a wide range of cancer types and plays an important regulatory role in tumor suppression. MRX34 is a double-stranded RNA oligonucleotide that has been incorporated into amphoteric liposomes and examined in different cancer types. Amphoteric liposomes increase the circulation time of MRX34 in the blood stream and deliver higher amounts of it to target tissues. Immune-related adverse reactions associated with MRX34 in 2016 limited its application. However, in 2017, the first in-human phase I clinical study showed that MRX34 with dexamethasone premedication had acceptable safety and tolerability in patients with advanced solid tumors. In this study, MRX34 was injected twice weekly for 3 weeks and showed anti-tumor activity in a subset of patients^173^. However, we still need an efficient carrier for miR-34 that avoids immune activation during delivery to tumors^174^.

Correspondingly, Miravirsen, a LNA-based antisense oligonucleotide to miR-122, is nontoxic even in humans and has successfully completed Phase II clinical trials. Miravirsen is being developed by Santaris Pharma for hepatitis C treatment^60,175^. An LNA-based anti-miR-155, MRG-106, has shown tolerability and pharmacokinetic benefits in patients with cutaneous T-cell lymphoma in a first-in-human phase I clinical trial^176^. Additionally, MesomiR 1 is a bacterially-derived small spherical minicell designed to deliver double-stranded 23-base-pair miR-16 mimics to pleural mesothelioma *in vivo*. MesomiR 1 has now passed the phase I clinical study due to its safety and tolerability, and entered a phase II clinical trial^177,178^. MRG-201, a LNA-miRNA mimic, which restores miR-29b in human cancer, is currently undergoing phase II clinical trials *via* intradermal injection^171^. Despite the small number of clinical trial studies on miRNAs, many small RNAs have shown beneficial effects in tumor treatment that warrant their progression to clinical trials. Some of them, like miR-21, miR-182, miR-15a, miR-103/107, miR-122, miR-195, and miR-208, are now in preclinical studies and await further clinical investigations.

## Challenges and perspectives

Several lines of evidence demonstrate that miRNAs are emerging as promising noninvasive biomarkers and cancer therapies. MiR-based therapy is divided into 2 therapeutic strategies: modulation of miRNA biogenesis and miRNA replacement or antisense therapy^179,180^. A major advantage of miRNA replacement therapy is that a single small molecule is able to regulate several hundred targets in different oncogenic pathways^181^. Additionally, this approach can restore normal function in target cells with fewer side effects involving noncancerous tissues. Furthermore, the phenomenon of acquired resistance to therapy is a challenge in the field of cancer. Studies have shown that several miRNAs are involved in radiation-, immune-, and chemoresistance in various cancer types^20^. We envision that targeting miRNAs combined with other cancer therapy strategies could successfully increase treatment efficiency, overcome cancer resistance, and improve patient outcomes.

However, the translation of promising preclinical findings to the clinical setting faces technical challenges related to tolerance issues and the lack of a proper delivery system. To overcome these limitations, successful carriers of miRNA molecules must be designed to enhance bioavailabilities and the endosomal escape of particles, and to evade the host’s immune response^63,182^. So far, diverse miRNA delivery vehicles have been developed that have their own advantages and disadvantages. Some of these systems are currently being used in phase I and II clinical trials for gene therapies. Furthermore, it is likely that designing and choosing delivery systems and routes of administration based on the type and stage of cancer will accelerate clinical translation of miRNA-replacement therapies. For example, oral delivery can be helpful for cancers related to the digestive tract^183^. In addition, use of mucoadhesive chitosan-based nanoparticles is effective for oral administration of drugs. Less invasive intranasal administration can overcome the BBB and be used for targeting CNS diseases^184^; cell-penetrating peptide-mediated delivery systems will be useful for this purpose. Additionally, local delivery of miRNAs *via* viral vectors or nanofibers seems more efficient and less toxic in treating early-stage cancers, whereas for metastatic cancer, more stable and less immunogenic delivery vehicles with high affinity and specificity for cancer sites elicit better responses^63,185^. Furthermore, the use of “cocktail” carriers or multiple simultaneous approaches may increase the transfer efficiency of miRNAs to target cells. More recently, a report by Wang et al. reported that a let-7a core dendrimer encapsulated in natural killer cell-derived exosomes significantly inhibited neuroblastoma growth without inducing immune response^186^.

At the same time, to improve the therapeutic effectiveness of miRNAs, efforts should be made to optimize therapeutic doses and schedules based on personalized medicine. Moreover, due to intratumor heterogeneity, targeting multiple molecules or pathways simultaneously with the goal of synergic effects will likely prove more efficient. In this regard, manipulation of different miRNAs in the same cells would be a more promising strategy to fully eradicate certain tumors.
